# From DOTS to the Stop TB Strategy: DOTS coverage and trend of tuberculosis notification in Ebonyi, southeastern Nigeria, 1998-2009

**DOI:** 10.4314/pamj.v9i1.71187

**Published:** 2011-05-31

**Authors:** Kingsley Ukwaja, Isaac Alobu, Ngozi Ifebunandu, Chijioke Osakwe, Chika Igwenyi

**Affiliations:** 1 Department of Internal Medicine, Ebonyi State University Teaching Hospital Abakaliki, Ebonyi State, Nigeria; 2 National Tuberculosis and Leprosy Control Program, Ministry of Health, Ebonyi State Nigeria; 3 Department of Medicine, Federal Medical Centre Abakaliki, Nigeria; 4 World Health Organisation, Southeast zone, Nigeria

**Keywords:** Tuberculosis, Epidemiology, control, DOTS, case finding, Nigeria

## Abstract

**Background:**

Nigeria ranks fourth among the 22 high tuberculosis (TB) burden countries. The estimated incidence of all TB cases in 2009 was 311/100,000 population. Since the implementation of DOTS in Ebonyi state, southeast Nigeria, the epidemiology of TB in the region has not been documented. Therefore, the objective of this study was to assess the type and case notification dynamics of TB following DOTS expansion and to examine age- and sex-specific trends in TB notification rate.

**Methods:**

A retrospective trend analysis of case notification data from the Ebonyi State Ministry of Health records from 1998 to 2009 was conducted. Patients were diagnosed according to the National TB and Leprosy Control Programme guidelines. Denominators for TB notifications were derived from population census data.

**Results:**

Of the 24, 475 cases notified between 1998 and 2009, 66% were smear-positive, 31% smear-negative and 3% had extra-pulmonary tuberculosis. Overall, the proportion of new smear-positive cases notified decreased continuously from 67% to 48% in 2009 while that of smear-negative cases increased from 29% to 40% in 2009. In 2005, 13 (100%) of the local government areas were covered by DOTS. Despite initial increase in case notification with DOTS expansion, the case notification rate had a mean annual decline of 3.1% for all TB cases (falling from 123/100 000 to 77/100 000), and of 5% for smear-positive patients (falling from 80/100 000 to 32/100 000). Smear-positive notification rate in children <14 years was consistently low while 25-34-year-old persons were affected most. However, smear-positive rates among persons aged =65 years did not change. Overall, annual new smear-positive notification rates were persistently lower in females than males.

**Conclusion:**

TB notification rate shows a decreasing trend in our region with a pool of infectious cases in young-persons. Additional targeted, type and age-/sex- specific interventions for TB control are needed.

## Background

Tuberculosis (TB) remains a leading infectious cause of death amongst adults in Sub-Saharan Africa. The situation is fuelled by extreme poverty, weak political will, inadequate human resource, weak health systems, inadequate financing of control measures, poor laboratory services, drug-resistant TB; and, in the last two decades, due to the HIV/AIDS pandemic [1]. In 1993, the World Health Organization (WHO) declared tuberculosis a global emergency and introduced the directly observed treatment, short course (DOTS), strategy for global tuberculosis control [2]. Achieving the DOTS strategy is believed to be the most effective route to TB control. Thus, the total number of countries implementing DOTS increased steadily from 1995 to 2003, and has since remained stable at around 180 countries [3].

Studies from resource-poor settings in Asia had demonstrated that DOTS is effective for TB control [4, 5]. But, DOTS strategy has produced variable success in Africa with some experts worried that the strategy has failed [6, 7]. With estimated new cases of TB at 460, 000 in 2009, Nigeria ranks fourth among the 22 high TB burden countries in the world [8]. The Nigerian National Tuberculosis and Leprosy Control Programme (NTBLCP) have adopted the DOTS strategy and 99% geographic coverage had been achieved by 2008 [8, 9]. Existing data on the population-level notification rate for TB in Nigeria are lacking, those available are derived largely from hospital data [10, 11]. Such data are often incomplete and do not define the relationship within individual populations bearing the brunt of disease. Insights on the performance of the DOTS strategy in populations can be derived from case-notification dynamics in well-defined populations [7].

Although DOTS has been implemented in Ebonyi State, Southeast Nigeria since 1996, its performance, including trends in TB cases notified has not been evaluated. Also, although The WHO – African Region: Strategic plan for TB Control for the African region: 2006 – 2010 [1], recommended that the trend of tuberculosis disease be determined in high burden African countries by 2010, we found no population-based study on the trend of TB notification in Nigeria. We therefore, investigated the impact of the implementation and expansion of DOTS on the epidemiological changes in TB notifications in Ebonyi state, during 1998 – 2009. The study objective was to document the case notification and type of TB dynamics following DOTS expansion, and to examine age- and sex-specific trends in TB notification rate in the state. Such information may provide a scientific basis for any modification of present TB – control strategies.

## Methods

### Study setting

Ebonyi state is located in the southeast geopolitical zone of Nigeria with an estimated population of over 2.5 million people [12]. The state has 13 local government areas (LGA) with 130 health care facilities currently providing DOTS services. All treatment units have standard unit registers from the NTBLCP. Each LGA have a Tuberculosis and Leprosy (TBL) control supervisor responsible for managing and coordinating TB and leprosy control activities as well as keeping up-to-date and accurate record of activities of TB and leprosy control activities in the LGA. They also provide monthly report to the State TBL control officer whose responsibilities among others include collection, collation and analysis of data on leprosy and TB activities in the state and dissemination of reports to the Federal Ministry of Health as well as other organizations as appropriate.

The NTBLCP uses the WHO-recommended recording and reporting guidelines and forms for monitoring and evaluation of programme activities. Briefly, patients’ with clinical features suggestive of TB are screened for confirmation of the diagnosis and initiation of treatment. Pulmonary tuberculosis (PTB) suspects submit three sputum samples (spot-morning-spot). Smears are graded according to the guidelines of the International Union against Tuberculosis and Lung Disease and to the recommendations of the National Workers Manual [9] Patients with at least two positive smears are considered smear-positive and those with three negative smears are treated with antibiotics and then re-evaluated. The diagnosis of extra-pulmonary TB (EPTB) is usually made clinically by a medical officer [9].

### Design and data collection

We undertook a retrospective trend analysis of case notification data notified by each of the LGA to the National Tuberculosis and Leprosy Control Programme office, Ministry of Health, Ebonyi State during 1998-2009. The quarterly reports reviewed contained basic information such as patient′s age, sex, treatment category, and TB type.

### Statistical analysis

The collected data were recorded and analysed using Microsoft Excel Templates for the analysis and presentation of tuberculosis epidemiologic data provided by the WHO TB Epidemiology and Surveillance Virtual Workshop [13]. Denominators for rate calculations were based on extrapolations of the 2006 population census data, assuming a linear trend in age- and sex-specific distributions over the study period. The outcome variables were DOTS coverage (number of DOTS centre), number of cases and type of TB notified yearly, the case notification rate, and the age- and sex-specific trends in TB notification rate.

### Ethical Approval

Ethical approval was not required as the survey was based on retrospective data and this report is part of standard public health practice. Permission to publish this report was granted by the National Tuberculosis and Leprosy Control Programme office, Ministry of Health, Ebonyi State, Nigeria.

## Results

Over the 12-year period from 1998 to 2009, 24 475 patients with all forms of TB were notified. Of these, 66% were smear-positive, 31% smear-negative and 3% EPTB. Although the proportion of smear-positive and smear-negative cases overall varied during the period; the proportion of new smear-positive cases decreased consistently from 67% in 2003 to 48% in 2009. And, the proportion of smear-negative cases increased from 23% in 2003 to 40% in 2009 (Table 1). The proportion of extra-pulmonary TB notified remained consistently below 3% but during 2007 to 2009 it has risen to 4 – 5%. Similarly, the proportion of relapses, treatment failure, return-after-default, and others remained below 5%.

**Table 1:** Trend of tuberculosis cases notified by type and proportion in Ebonyi, Southeast Nigeria from 1998 to 2009

	1998	1999	2000	2001	2002	2003	2004	2005	2006	2007	2008	2009
new Sm+	66%	65%	65%	63%	64%	67%	60%	57%	55%	51%	50%	48%
Sm –	27%	26%	27%	29%	27%	23%	30%	32%	35%	37%	39%	40%
EPTB	3%	3%	2%	3%	2%	3%	3%	3%	3%	5%	4%	5%
Relapse	1%	2%	1%	2%	2%	2%	2%	1%	1%	1%	2%	2%
Failure	–		0%	0%	1%	0%	1%	1%	1%	0%	0%	0%
RAD	–		2%	3%	4%	5%	3%	4%	3%	1%	1%	1%
Others	3%	4%	3%	–	–	0%	1%	2%	2%	5%	4%	4%

EPTB: diagnosis of extra-pulmonary Tuberculosis, Sm: smear, RAD: return-after-default

Although the case notification rate varied initially; there was a mean annual decline of 3.1% for all TB cases notified (falling from 123/100 000 in 2000 to 77/100 000 in 2009) (Table 2), and of 5% for smear-positive patients (falling from 80/100 000 in 2000 to 32/100 000 in 2008) (Table 2). Also, although the total number of TB cases notified yearly varied during the period (1809 – 2312), the increase in TB cases notified correlated with the decentralisation and expansion of the DOTS services to more health facilities, which increased from 17 in 1998 to 102 in 2009 (R2 = 0.63).

**Table 2:** Case-notification rates for all new; and new smear positive tuberculosis cases in Ebonyi, Southeast Nigeria, 1998 to 2009

	Number of		Number of	
		
Year	all new cases	CNRA/100, 000	new Sm+ cases	CNRS+/100, 000
1998	1967	111	1308	74
1999	2198	121	1429	78
2000	2312	123	1496	80
2001	2119	110	1341	69
2002	2101	106	1348	68
2003	1999	98	1312	64
2004	2081	99	1247	59
2005	2079	96	1193	55
2006*	2013	93	1105	51
2007	1925	86	985	44
2008	1872	81	742	32
2009	1809	77	872	37

* 2006 census population = 2, 176 947; Estimated annual growth rate = 2.8%; TB = tuberculosis; CNRA: Case notification rate for all forms of TB/100 000 population; CNRS+ = case notification rate, for smear positive TB cases/100 000 population; Sm+ = smear positive

**Figure 1:**
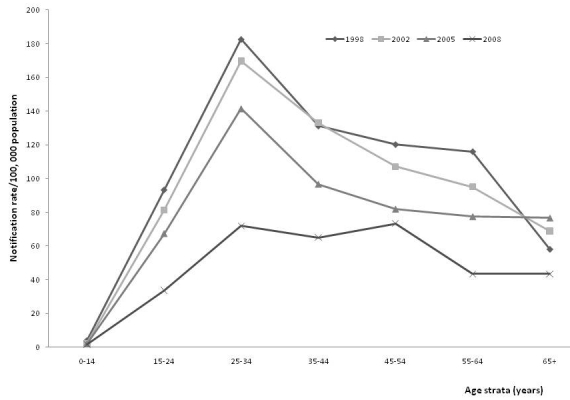
Notification rate for all new smear-positive tuberculosis by age-strata, 1998-2008, Ebonyi Southeast, Nigeria

Comparison of national census data from 1988 with data obtained in 2006 showed that the age-sex composition of the population had not changed significantly. Subsequent analysis of the age-specific distribution of TB notifications showed that the rate of smear-positive TB was low in the 0–14 age groups, but increased in the 15–24, and 25–44 years age groups. However, in the group of 65-year-olds or over, the rate leveled off (Figure 1). Age-dependent TB rates also showed a decline over time, but with a constant relative peak in the 25–34 years age group (Figure 1). In 2008, the 25–34 and 45–54 years age group had the highest notification rates.

**Figure 2:**
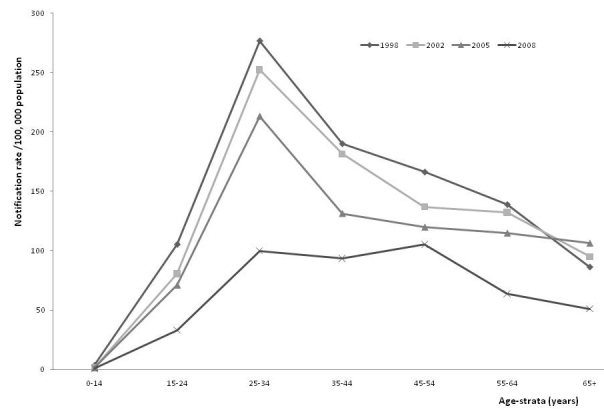
Notification rate for new male smear-positive tuberculosis by age-strata, 1998 – 2008, Ebonyi Southeast, Nigeria

**Figure 3:**
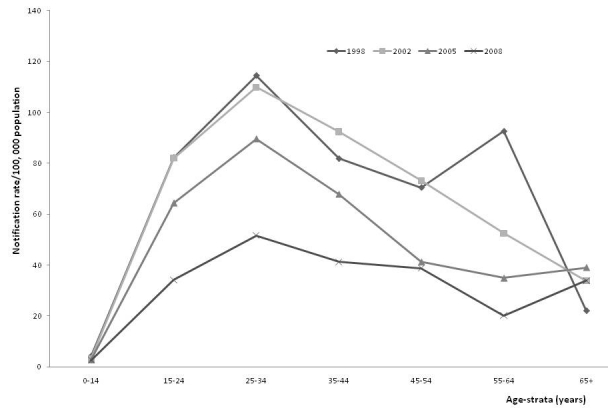
Notification rate for new female smear-positive tuberculosis by age-strata, 1998 – 2008, Ebonyi Southeast, Nigeria

Overall, more smear-positive cases were notified in males than females with peaks in 1998 of 277/100 000 (male population) and 115/100 000 (female population) respectively. In males, the 25–34 years age groups had the highest smear-positive notification rates, but in 2008, smear positive notification rates were also high in the 45–54 years age group before a decline in older patients (Figure 2). Similarly, in females, the 25–34 years age groups had the highest smear-positive notification rates before a relative decrease over time in older females (Figure 3).

## Discussion

One of the major aims of a TB control programme is to notify as many cases as possible. Implementing the DOTS strategy is believed to be a key strategy to achieving this goal [14]. The present study shows that, with the expansion of DOTS services, there was an overall increase in the number of TB cases notified. The State Tuberculosis and Leprosy Control Programme (STBLCP) and the German Leprosy Relief Association initially introduced DOTS in only a few LGAs following this by systematic scale-up, introducing more LGAs and public health facilities into the programme every year. Full LGA coverage was achieved in 2005 – nine years after the creation of the state and introduction of DOTS in the region. Although some newly constructed clinics and most private health facilities in the state are yet to be reached by the programme, this stepwise scale-up is critically important in ensuring judicious utilization of scarce resources and incorporating lessons learnt from the challenges that emerged in the course of expansion [14].

Unlike previous report from other African countries [6, 14, 15], as well as National figures for Nigeria which shows an increasing trend in all and smear-positive TB notification rate [3, 8], we observed a decreasing trend in all and smear-positive TB notification rate in Ebonyi since 1998. Much of the decrease probably reflects real changes in the incidence of TB in the region. But, this is surprising as the presence of the current HIV epidemic and other well-known factors influencing TB trends (poverty, malnutrition, and management failures in the treatment system) [16] in the state did not seem to alter this dynamics. This may be due to the usual problems of underreporting of TB because of problems with diagnosing TB in children, pregnant women, missed smear-negative patients and those with extra-pulmonary disease as well as systemic factors associated with access to TB care [15].

Also, in 2007, the WHO documented regional differences in the case notification rate for all and smear-positive TB in Nigeria [3]. The most likely reason for this variation is, the DOTS strategy was introduced in most states in the southern region more than a decade before it reached the northern region [17]. However, with the introduction and expansion of DOTS services in northern Nigeria from 2002, the region currently accounts for 55% to 65% of total TB cases notified annually in the country [17]. This systematic scale-up of DOTS services recently in northern Nigeria most likely accounts for the increasing trend in TB notification rate in the country. The 2007 case notification rate for all TB cases of 86/100,000 population observed in this study falls within the range (61/100,000 to 167/100,000) estimated by the WHO for the region where Ebonyi state is located in the same year [3].

In our study, we observed a progressive increase in the proportion of smear-negative pulmonary tuberculosis (PTB) during 2003 to 2009. This was associated with a concomitant continuous reduction in the proportion of smear-positive PTB. This trend in PTB cases had recently been documented nationally [18], and similar findings had been observed in Ethiopia [19]. The increasing number of smear negative PTB cases might be due to high proportion (about 32%) of HIV infection in TB in Nigeria [3]. Because of their compromised immune response and lack of formation of cavitations [20], HIV infected patients are twice more likely to have a smear-negative but culture positive PTB [21]. The trend in the proportion of extrapulmonary tuberculosis (EPTB) observed was lower than in other studies [14, 20, 22], but the proportion has gradually increased over the 12-year period. This might suggest that more cases of EPTB are being managed in specialist centres and it also indicates that the control programme should strengthen the reporting of these cases.

A key epidemiologic observation associated with a decreasing incidence of TB is usually an increased proportion of cases in older patients [22].This was particularly dramatic after the introduction of chemotherapy in the 1950s, when transmission of the Mycobacterium to the young was significantly reduced [22, 23]. As previously documented in Turkey [22], we observed a low incidence in smear-positive TB in children (7,22], the incidence then increased to peak in the 25-34 year olds before a subsequent decline. This indicates that in our region, there is a pool of infectious TB cases in young people. Thus, the programme should target this group for early case detection and treatment in order to interrupt transmission, reduce morbidity / mortality and prevent the emergence of drug resistance.

Furthermore, smear-positive TB cases commonly infect children and the young in populations with a high risk of TB infection. Since they have been infected before; the middle- age and older-age groups, are protected against exogenous infection. Thus, following infection with TB, the risk of developing the disease within 5 years is high, leading to accumulation of cases at 10–15 years of age [22]. Our study suggests that most of the infection of our patients occurred during late childhood and early adolescence. When TB disease and transmission are controlled, age at disease occurrence increases first to middle-aged, then to the older individuals [22]. Therefore, it could be indirectly said that the risk of TB infection in our region remains high.

Compared to males, the proportion of female smear-positive cases notified was found to be consistently low, and it was exceptionally lower among patients older than 45 years. This may indicate a genuine gender differences in TB epidemiology. The reasons for these differences have been issues for discussion and debate, and are likely to result from various factors, including access to care, ethnicity, the influence of the HIV co-epidemic, as well as other biological, social and cultural variables [24]. Probable reasons attributed to lower TB infection, and hence notification in women include higher immune responses which have been found to be more vigorous in women, resulting in greater antibody production and increased cell-mediated immunity after immunization [24, 25]. The role sex hormones play in the modulation of immune responses needs to be explored in further studies. Nevertheless, a recent Pakistani study showed that after adjusting for demographic and socio-economic differences; more female smear-positive cases were notified in poorer districts than males [26].

Some limitations of our analysis should be kept in mind. As our review was based on quarterly reports retained at the State Ministry of Health, we cannot exclude the possibility of poor recording and reporting systems. However, this has been reduced via several recording and reporting training and re-training organized by the State TB and Leprosy Control Programme for staff working in the public and private health sectors. Internal quality assurance of the data collected over time has been maintained by ensuring that all the LGAs collect their data using standardized TB report forms and registers from the WHO/NTBLCP. Similarly, external quality assurance have been maintained via monthly field visits by the State TBL control officer to DOTS sites in the LGAs to collate the data and monitor data collection in the site. The collected data are further verified during quarterly meetings of the State TBL control officer and all the local government TBL supervisors. Also, due to systemic factors associated with access to care, it is possible that the case notification rates of TB in the state are underestimated.

## Conclusion

In conclusion, TB case notification rates tend to decrease in Ebonyi state. A characteristic epidemiological feature associated with this decrease has been a concomitant increase in the proportion of smear-negative cases compared to smear-positive PTB patients. Infectious smear-positive PTB patients remain highest in the young age-group irrespective of gender but notification rates in females are consistently lower. Available data concerning trends in TB are very important in Nigeria for detecting changes in epidemiological patterns. The NTBLCP should target the young age-groups in improving case finding and interrupting transmission of TB. Also, efforts should be made in improving the recording and reporting of smear-negative TB and EPTB cases in the region.
